# Diabetes and the Metabolic Syndrome: Possibilities of a New Breath Test in a Dolphin Model

**DOI:** 10.3389/fendo.2013.00163

**Published:** 2013-11-25

**Authors:** Michael Schivo, Alexander A. Aksenov, Laura C. Yeates, Alberto Pasamontes, Cristina E. Davis

**Affiliations:** ^1^Department of Medicine, Division of Pulmonary, Critical Care, and Sleep Medicine, University of California, Davis, Sacramento, CA, USA; ^2^Center for Comparative Respiratory Biology and Medicine, University of California, Davis, Davis, CA, USA; ^3^Department of Mechanical and Aerospace Engineering, University of California, Davis, Davis, CA, USA; ^4^National Marine Mammal Foundation, San Diego, CA, USA

**Keywords:** bottlenose dolphins, metabolic syndrome, diabetes, exhaled breath, volatile organic compounds

## Abstract

Diabetes type-2 and the metabolic syndrome are prevalent in epidemic proportions and result in significant co-morbid disease. Limitations in understanding of dietary effects and cholesterol metabolism exist. Current methods to assess diabetes are essential, though many are invasive; for example, blood glucose and lipid monitoring require regular finger sticks and blood draws. A novel method to study these diseases may be non-invasive breath testing of exhaled compounds. Currently, acetone and lipid peroxidation products have been seen in small scale studies, though other compounds may be significant. As Atlantic bottlenose dolphins (*Tursiops truncatus*) have been proposed as a good model for human diabetes, applications of dietary manipulations and breath testing in this population may shed important light on how to design human clinical studies. In addition, ongoing studies indicate that breath testing in dolphins is feasible, humane, and yields relevant metabolites. By studying the metabolic and cholesterol responses of dolphins to dietary modifications, researchers may gain insight into human diabetes, improve the design of costly human clinical trials, and potentially discover biomarkers for non-invasive breath monitoring.

Diabetes mellitus is a chronic human disease with large economic and medical impact. A median of 8.2% of the US population has type-2 diabetes (DM2), which is thought to develop from a complex interplay between genetics, diet, obesity, and potentially other common illnesses such as obstructive sleep apnea (1–5). Often diabetes develops in humans together with elevated triglycerides. In fact, the combination of abdominal obesity, lipid abnormalities (e.g., low HDL and/or elevated triglycerides), elevated blood pressure, and/or abnormal fasting glucose levels that do not reach the diagnostic levels for DM2 constitute the metabolic syndrome (MetS) (6, 7), though exact definitions and prevalence estimates of the MetS vary according to published guidelines (8–11). It is useful to think of the MetS and diabetes as existing along a spectrum, and both are directly related to the development of cardiovascular disease. Diabetes care is estimated to comprise up to 14% of gross US healthcare expenditures (1), and much of this cost is driven by complications of DM2 including cardiovascular disease, stroke, kidney disease, retinopathy, and foot ulcers (12). DM2 and the MetS have reached epidemic proportions and without timely interventions, costs and morbidity are expected to rise (13).

Though an abundance of human clinical data regarding DM2 exists to guide therapy, much of the understanding of how therapies work remains elusive. For example, recent attention has focused on dietary effects on DM2 and the MetS. It is known that Mediterranean diets (i.e., those rich in monounsaturated fats such as olive oil, legumes, lean meats, and vegetables) may improve insulin sensitivity, obesity, cholesterol regulation, and glycemic control, among other measures (14–16). The antioxidants found in olive oil may have anti-inflammatory and anti-hypertensive effects (17), however, no single component of the Mediterranean diet studied in isolation has shown similar outcomes as the diet as a whole. Moreover, many of the studies looking at the Mediterranean diet differ in their composition making generalization of the diet problematic. Other diets, such as low-carbohydrate and low-fat, have also been studied in regard to their impacts on components of DM2 and the MetS with variable results (18). Clearly, an improved understanding of diet is important as one would expect dietary interventions to be among the most cost-effective ways to control DM2 and the MetS.

In addition to the problem of understanding therapeutic interventions in DM2 and the MetS, current monitoring of these complex diseases requires, in part, invasive blood testing to assess glucose and lipids. One novel and emerging method of research which may benefit several lines of human clinical investigation, including to the study of DM2 and the MetS, is exhaled breath analysis. Exhaled breath analysis has been used for centuries in human disease. For example, volatile ketones resulting from the lipolysis of adipocytes when insulin production is diminished (e.g., type I diabetes) or in starvation states can be smelled on the breath as a sweet, fruity odor (19). More recent medical uses of exhaled breath include cancer detection, lung diseases, aging, and gastrointestinal disorders, to name a few (20, 21).

## Exhaled Breath for MetS and DM2 Monitoring

Exhaled breath is collected as an ambient gas or liquid [exhaled breath condensate (EBC)] using non-invasive collection systems that cool expired breath, and both of these fractions contain interesting metabolites. The advantage of analyzing the gaseous fraction is the identification of volatile compounds which may be conducted in real-time. Such volatile compounds are likely representative of end-points of metabolic pathways. Conversely, the advantage of EBC is the capture of higher molecular weight, non-volatile compounds such as peptides and lipids, or even particles such as viruses trapped in exhaled droplets. There is a wide range of analysis platforms and computational strategies to interpret the vast amount of information present in exhaled breath, and many of these platforms are scalable to permit point-of-care use (22). Exhaled breath, whether collected in gas or liquid form, represents a novel matrix of human and animal data which may ultimately serve as a complementary or stand-alone fluid for disease monitoring.

Of significance to diabetes, non-invasive breath testing represents an attractive way to test for glycemic control. Current recommendations by the American Diabetes Association include multiple daily blood glucose testing in diabetics on insulin, and less frequent testing in diabetics on oral therapy (23). Current blood glucose tests are uniformly invasive as they require finger sticks at a minimum. Potential benefits to breath testing include avoidance of painful finger sticks, improved compliance with glucose monitoring, and the potential to assess insulin levels, which is currently an unavailable point-of-care test for most diabetics (24). Better control and early diagnosis of diabetes through non-invasive means may help lower morbidity and mortality.

There is a paucity of studies assessing breath biomarkers in human diabetes, though important efforts have been made. Greiter et al. were able to distinguish 21 insulin-dependent DM2 subjects from 26 healthy controls using proton-transfer reaction mass spectrometry (PTR-MS) analysis of exhaled gas (25). Though many of the chemicals were not identified, compounds including acetone, dimethyl sulfide, isoprene, butanol, and pyridine were identified. In this study, only acetone was seen in significantly elevated levels in DM2 subjects on exogenous insulin. Though this is surprising in DM2 as ketogenesis and acetone production are typically thought to arise in type-1 diabetes, breath testing may be able to uncover compounds that challenge the paradigm of diabetes understanding. Guo et al. sought to correlate human breath acetone concentrations to blood glucose levels (26). In this study, the correlation was not robust, but the results are promising. In a study of 16 subjects undergoing an oral glucose tolerance test, elevated exhaled acetone levels were able to classify diabetics from non-diabetics, though there were large inter-individual differences (27). Goerl et al. studied the breath of 30 subjects with advanced kidney disease undergoing dialysis (28). In the subset of subjects with DM2, there was significantly less variation in longitudinal breath acetone concentrations. Landini and colleagues studied breath acetone production in patients undergoing medically supervised, calorie-restricted weight loss (29). They showed that acetone concentrations were elevated in subjects with more weight loss, presumably reflecting a starvation ketosis rather than a lack of insulin. Though this study did not specifically assess diabetics, it supports the principle of acetone evolution in breath as a reflection of a systemic metabolic process.

Recently, ^13^C-labeled glucose has been studied as a non-invasive breath measure of glucose metabolism and insulin resistance in DM2. Mizrahi et al. demonstrated good correlations between serum glucose (positive) and insulin (negative) levels to ^13^C-glucose breath levels after ingestion of ^13^C-glucose in healthy volunteers (30). Another small study correlated breath ^13^C-glucose to both urban and rural Asian Indian populations in terms of insulin resistance, body mass index, and waist circumference (31). Jetha et al. assessed the role of non-invasive breath ^13^C-glucose monitoring in obese children where fears of needle-testing for glucose levels are high (32). As with prior studies, they found significant correlations between the breath and serum tests. These studies indicate that ^13^C-glucose tests and acetone concentrations may be important non-invasive measures of glucose metabolism in DM2. Whether acetone is important alone or in combination with other, undiscovered biomarkers remains to be seen.

Related to the MetS and lipid metabolism disorders, few non-invasive breath studies have been published. The metabolism of dietary constituents is routinely assessed using exhaled CO_2_, though this is a rough marker of lipid metabolism since the metabolism of carbohydrates and proteins produce CO_2_ as well (33, 34). Indirect evidence of lipid metabolism comes from the breath evolution of lipid peroxidation products such as ethane and pentane (35), though these may be due to general or airway inflammation and not to lipid balance. A small study in spontaneously overweight mice measured increased exhaled ^13^CO_2_ as a marker of increased lipolysis when the mice were given a thyroid hormone analog (36). This suggests that CO_2_ may be a suitable measurement in certain experimental settings.

## Barriers to Breath Analysis in the MetS and DM2

As with many novel techniques, breath analysis must overcome several barriers before it is accepted as a part of clinical disease management. There are certainly success stories such as exhaled nitric oxide for use in allergies and asthma (37), but the majority of breath tests, particularly for the MetS and DM2, are still experimental. First, few large-scale studies have been conducted to validate the findings from smaller studies. This creates substantial problems with technique validation and application across populations. Next, the validation of individual or groups of biomarkers (i.e., acetone, glucose, etc.) requires large amounts of subjects across a range of ethnic and geographic groups. Nomograms for many of the compounds identified in DM2 and the MetS simply do not exist. In addition, these biomarkers would need to be assessed longitudinally to account for fluctuations over time, season, and disease activity. Finally, costs associated with the development of breath analysis technologies may be high, but as components become refined and produced to scale, costs would be expected to decrease (think of standard glucose meters). The bottom line is that more rigorous experimentation is needed to validate and advance breath testing in the MetS and DM2.

## A Dolphin Model of the Human MetS

In this issue and previously, Venn-Watson and colleagues have reported that Atlantic bottlenose dolphins (*Tursiops truncatus*) are a good model of the human MetS and insulin resistance (38, 39), though accepted definitions of the human MetS vary somewhat from that used by the authors (6–9). In 2011 the authors published a study where managed dolphins were fed dextrose or Spanish mackerel (a high-protein, low-carbohydrate meal) (38). Both meals resulted in sustained hyperglycemia: up to 10 h post-dextrose and 5 h post-mackerel. Accompanying insulin levels were also elevated which is similar to the hyperinsulinemia seen in human DM2 as a result of impaired peripheral insulin metabolism (40). The authors’ 2007 work demonstrated that serum glucose was elevated in fasting dolphins, and that fasting platelets, gamma-glutamyl transpeptidase, and alkaline phosphatase were also significantly elevated (39), as may be seen in the human MetS (41, 42). In the present work, the authors further describe the metabolic abnormalities among managed and free-ranging dolphin populations. In addition to elevated insulin levels, affected dolphins in the managed group also had elevated glucose, total cholesterol, HDL-C, VLDL-C, iron, transferrin saturation, and unmodified adiponectin. These elevations mirror some of the changes seen in humans with the MetS. Interestingly, managed dolphins with aberrant blood markers do not appear to have an elevated mortality, at least not attributed to their metabolic dysfunction (43). The aforementioned body of work suggests that dolphins share similar physiologic responses with human diabetics in reference to fasting and bolus-feeding states, but that dolphins may utilize diet fluctuations and glycemic changes to non-pathologic ends. Thus, bottlenose dolphins may be a unique, phylogenetically close, and accessible model for human diabetes.

Importantly, the bottlenose dolphin model of DM2 and the MetS has a few key potential applications. First, hyperglycemia in dolphins appears to be a naturally occurring response to both fasting (39) and post-prandial states (38). As hypothesized, this may be an adaptive response to stress (e.g., starvation or intermittent food availability) to keep blood glucose available to the dolphins’ larger brains. Presumably dolphins naturally feed continuously in food-plentiful environments and therefore maintain more stable glucose and insulin levels, especially if they have a varied diet of fish.

As mentioned, the understanding of dietary influences on DM2 and the MetS is essential. Patterns and timing of food consumption have long been recognized as important to weight control in humans (44, 45), though longitudinal datasets demonstrating optimal feeding times in diabetic patients are limited to small studies of fairly short duration. One study that randomized women with DM2 and polycystic ovarian syndrome to higher breakfast versus dinner caloric intake for 90 days noted reduced levels of post-prandial glucose and insulin in patients who ate higher calories earlier in the day (46). This is supported by other evidence that consuming high calorie foods with a high glycemic index late in the day results in higher glucose levels and insulin resistance (47). Dietary manipulations in managed bottlenose dolphin populations could augment existing human data and provide insight to optimal feeding regimens (both in diet content and timing) that affect post-prandial glycemic and insulin indices in humans. Specifically, altering protein and carbohydrate ratios to a group of dolphins that “behave” like human diabetics without the associated complications is an attractive prospect.

Another important use of a bottlenose dolphin model for human disease lies in studying cholesterol fluctuations with dietary modifications. In the study by Venn-Watson et al. in this issue, all cholesterol components were elevated in dolphins with increased glucose and insulin levels. However, they were elevated in different ways than found in humans with diabetes as the HDL-C component was disproportionately elevated in dolphins. In humans, *low* HDL-C levels often associate with DM2, and therapy aims to increase the HDL-C component as it is considered cardio-protective (48). For example, in a case-control study assessing 113 humans with high HDL-C compared to 212 with low HDL-C on the incidence of DM2, 1.8 versus 21.7% (high HDL-C versus low HDL-C) developed DM2 (49). Though dolphins develop dyslipidemias as do humans, the mechanisms by which they develop them and, importantly, the fact they do not appear to have increased mortality compared to humans, could shed light into strategies to bolster HDL-C in humans. Further research into how dolphins maintain high HDL-C in the face of other metabolic imbalances could prove useful to understanding human lipid regulation.

As dolphins represent an appealing model of human DM2 and the MetS, and cetacean research should ideally serve the dual purpose of benefiting animal and human health, researchers may access both wild and managed dolphins. Two outstanding examples of successful programs where researchers study wild dolphin populations include the Mote Marine Laboratory in Sarasota, FL, USA, and Dolphin Research Australia based in New South Wales. Free-ranging dolphin research is informative, semi-natural, and externally valid, though it requires extensive effort (50). Conversely, managed dolphins are attractive in that more rigorous experimentation is possible; however, studies on these populations may not be applicable to wild dolphins due to differences in life conditions. Importantly for research, managed dolphins receive excellent care, have low mortality rates, and have annual survival rates of 99% (43, 51). Regardless of the population studied, efforts to optimize cetacean research and maximize data recovery are paramount. Specifically, one strategy may be utilization of exhaled breath.

## Exhaled Breath Evaluation of Dolphins

Though exhaled breath collection from cetaceans has not been rigorously evaluated, early work assessing exhaled nitric oxide has demonstrated feasibility (52). Exhaled breath analysis in dolphin populations has several potential advantages. Given that breath sampling techniques are non-invasive and do not obstruct the airway, one would expect breath sampling of dolphins to engender minimal concern. This would facilitate sample collection in both managed and wild populations. Anatomically, dolphins have respiratory systems which are separate from their gastrointestinal tracts. This prevents the oropharyngeal and gastrointestinal contamination of the collected breath which confounds collection and analysis in humans (53). Uniquely, cetaceans are explosive breathers, exchanging 70–90% of total lung capacity in 0.3 s (54). The sheer volume of a single dolphin breath is far greater than many human breaths in aggregate. This breathing behavior leads to rapid gas exchange, quick sample collection, and minimal contact time with the animals. In addition, bottlenose dolphins can hold their breath for about 7 min while diving, which leads to enrichment of the exhaled gas with endogenous metabolite compounds. Explosive breathing and breath-holding provide unique advantages to aid in the discovery of large amounts of exhaled compounds which may directly inform the design of human breath studies. Furthermore, the limited genetic variability in dolphins compared to humans would likely result in lower variability of the metabolic content in the exhalate. In a proof-of-concept study, we have been able to capture exhaled breath from bottlenose dolphins, and preliminary analysis indicates a chemical separation of fasting versus non-fasting animals based on breath constituents such as triglycerides (tentatively identified; unpublished data). As exhaled breath represents a novel and potentially robust matrix for disease detection, breath monitoring application in cetaceans appears logical and important.

In conclusion, DM2 and the MetS are important epidemics which are predicted to escalate in coming years. As diet is central to the development of, and potentially the control of, DM2 and the MetS, a focus on studying dietary influences is key. Cetacean models of DM2 and the MetS are interesting as Atlantic bottlenose dolphins (*T. truncatus*) have physio-chemically similar responses to alterations in diets as do humans, yet they do not seem to experience increased mortality. Also, the bottlenose dolphin is of closer phylogenetic relation to humans than many other animal models. Key differences in the regulation of HDL-C cholesterol alone are worth intensive scrutiny as novel mechanisms to bolster HDL-C levels in human diabetics may confer protection from cardiovascular disease. In addition, studies altering feeding intensity and diet composition in dolphins may have profound implications in human disease. The aforementioned advantages of breath analysis in dolphins present an ideal model for biomarker discovery in DM2 and the MetS. Further targeted analysis of some of the discovered biomarkers may facilitate application of breath analysis for DM2 monitoring tests in humans. The logical and ethical use of animal models to complement existing human data remains an important goal, especially as the cost and limited scope of human trials can benefit from as much guidance as possible. As we strive toward obtaining biological samples using the least invasive methods, exhaled breath as a novel matrix to study in both animals and humans emerges as a viable and interesting option. Though we are not ready to use exhaled breath analysis in lieu of invasive blood tests, this reality may not be far off.

## Conflict of Interest Statement

The authors declare that the research was conducted in the absence of any commercial or financial relationships that could be construed as a potential conflict of interest. The Guest Associate Editor, Stephanie Venn-Watson declares that, despite having collaborated with the author Cristina Davis, the review process was handled objectively and no conflict of interest exists.
